# Long-lasting complete response status of advanced stage IV gall bladder cancer and colon cancer after combined treatment including autologous formalin-fixed tumor vaccine: two case reports

**DOI:** 10.1186/s12957-017-1245-x

**Published:** 2017-09-11

**Authors:** Yuki Imaoka, Fumito Kuranishi, Tsubasa Miyazaki, Hiroko Yasuda, Tadao Ohno

**Affiliations:** 1 Department of Surgery, Innoshima Ishikai Hospital, 1962, Nakanosho, Innoshima, Onomichi, 722-7221 Japan; 2 Cell-Medicine Inc, 2-1-6-C-B-1, Sengen, Tsukuba, Tsukuba, Ibaraki, 305-0047 Japan

**Keywords:** Tumor vaccine, Gall bladder cancer, Colon cancer, Stage IV, Combined therapy

## Abstract

**Background:**

The prognosis of advanced (stage IV) cancer of the digestive organs is very poor. We have previously reported a case of advanced breast cancer with bone metastasis that was successfully treated with combined treatments including autologous formalin-fixed tumor vaccine (AFTV). Herein, we report the success of this approach in advanced stage IV (heavily metastasized) cases of gall bladder cancer and colon cancer.

**Case presentation:**

Case 1: A 61-year-old woman with stage IV gall bladder cancer (liver metastasis and lymph node metastasis) underwent surgery in May 2011, including partial resection of the liver. She was treated with AFTV as the first-line adjuvant therapy, followed by conventional chemotherapy. This patient is still alive without any recurrence, as confirmed with computed tomography, for more than 5 years.

Case 2: A 64-year-old man with stage IV colon cancer (multiple para-aortic lymph node metastases and direct abdominal wall invasion) underwent non-curative surgery in May 2006. Following conventional chemotherapy, two courses of AFTV and radiation therapy were administered sequentially. This patient has had no recurrence for more than 5 years.

**Conclusion:**

We report the success of combination therapy including AFTV in cases of liver-metastasized gall bladder cancer and abdominal wall-metastasized colon cancer. Both patients experienced long-lasting, complete remission. Therefore, combination therapies including AFTV should be considered in patients with advanced cancer of the digestive organs.

## Background

The prognosis of advanced cancer (notably late stage IV cancer) of the digestive organs remains very poor despite the advances in surgical treatment and chemotherapy, even with the latest immune checkpoint inhibitors (as discussed in the Educational Session: What’s next in Cancer Immunotherapy, American Society of Clinical Oncology, June 6, 2016). We have previously reported a case of advanced breast cancer with bone metastasis that was successfully treated [1] with combined treatments including autologous formalin-fixed tumor vaccine (AFTV) [2, 3]. Herein, we report the success of this approach in cases of advanced gall bladder cancer (stage IV, liver metastasis) and colon cancer (stage IV, abdominal and lung metastases).

## Case presentation

### Case 1

**Fig. 1:**
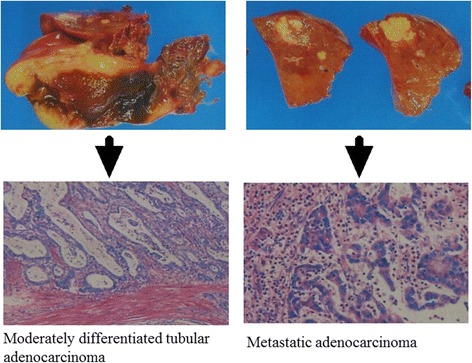
Histology of resected gall bladder carcinoma (case 1). Left side: moderately differentiated tubular adenocarcinoma in the gall bladder. Right side: liver-metastasized adenocarcinoma in S4 and S5

**Fig. 2:**
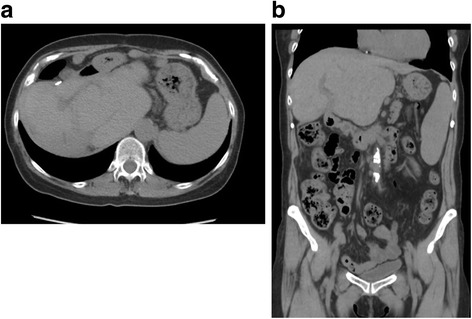
Computed tomography images of case 1. **a** and **b** No recurrence is noted

A 61-year-old woman with stage IV gall bladder cancer (T3N1M1; liver metastasis and lymph node metastasis) underwent surgery on May 9, 2011, including cholecystectomy, segmental resection (S4 and S5) of the liver, extrahepatic bile duct resection, lymph node resection, and anastomosis between the liver duct and the jejunum as an R0 resection. Histological analysis demonstrated moderately differentiated tubular adenocarcinoma and metastatic adenocarcinoma in S4 and S5 (Fig. 1). She was treated with AFTV (prepared as reported in [1], using paraffin-embedded autologous primary and liver-metastasized gall bladder cancer instead of breast cancer) as the first-line adjuvant therapy, followed by conventional chemotherapy, i.e., gemcitabine (800 mg: 6 courses, 1000 mg: 8 courses, 1200 mg: 16 courses) and titanium silicate-1 (TS-1; 80 mg/day) between October 2013 and April 2015. She has not shown any recurrence, as confirmed on computed tomography (CT), for more than 5 years (Fig. 2a, b).

### Case 2

**Fig. 3:**
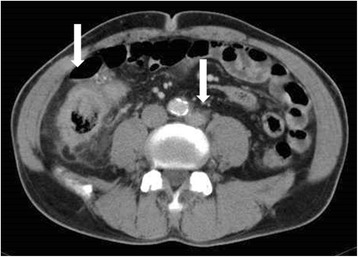
Computed tomography of case 2. An image before the operation shows para-aortic lymph metastasis and abdominal wall invasion (arrow)

**Fig. 4:**
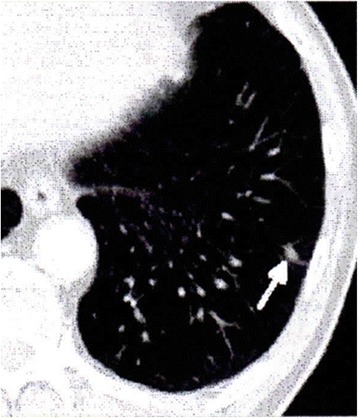
Computed tomography of case 2. An image before formalin-fixed tumor vaccine therapy shows a mass in the left lung (arrow)

**Fig. 5:**
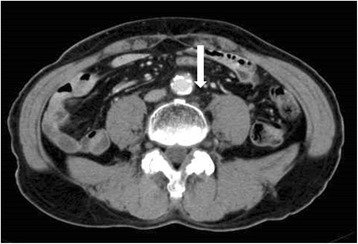
Computed tomography of case 2. An image shows disappearance of the para-aortic lymph metastasis after formalin-fixed tumor vaccine therapy (arrow)

**Fig. 6:**
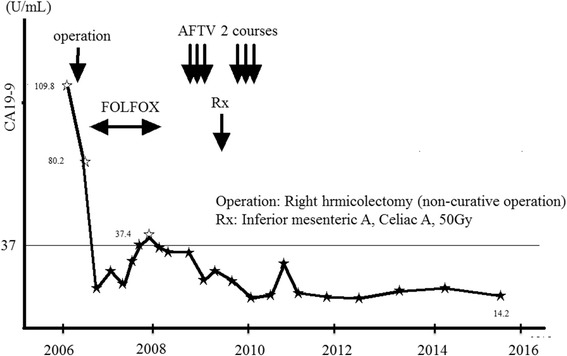
Change in the CA19-9 level. The CA19-9 level shows a gradual decrease. Operation: right hemicolectomy (non-curative operation). Rx: radiation therapy with inferior mesenteric artery to celiac artery (50 Gy)

A 64-year-old man with stage IV colon cancer underwent right hemicolectomy on May 12, 2006. As he did not visit a hospital, he was not aware of the symptoms until a very late stage of colon cancer. During surgery, he was diagnosed as T4b, N3, H0, P1, M1, with 11 metastases of 28 para-aortic lymph nodes and direct abdominal wall invasion accompanied by ascites (Fig. 3) and he underwent non-curative (R2) resection. He received first-line adjuvant chemotherapy (tegafur 400 mg/uracil 75 mg; 8 courses) until February 2008. However, the therapy was subsequently replaced with bevacizumab in combination with oxaliplatin, fluorouracil, and leucovorin (FOLFOX4: 13 courses) owing to an increase in the size of the remaining para-aortic lymph nodes. From May 2008, he was administered the third round of adjuvant chemotherapy comprising 12 courses of capecitabine (300 mg/day). However, a new 4-mm mass appeared on CT of the left lung in August 2008 and was confirmed in July 2009 (Fig. 4). Chemotherapy was continued until he experienced cerebral infarction in February 2009. After rehabilitation, he was administered AFTV (prepared as reported in [1], using paraffin-embedded autologous primary and lymph node-metastasized colon cancer instead of breast cancer) in August 2009, and he received radiation to the increasing para-aortic lymph nodes (50 Gy/25 frac/5 weeks). After the radiation therapy, he was administered a second course of AFTV in July 2010. The CA19-9 level decreased gradually, and the para-aortic lymph node metastases disappeared (confirmed in 2015; Fig. 5 for the lymph node and Fig. 6 for the CA19-9 level). The mass observed in the left lung also disappeared without direct radiation therapy. Combined treatments with conventional chemotherapy, two courses of AFTV, and radiation helped to resolve his condition, and there has been no recurrence for more than 5 years.

## Discussion and conclusions

Gall bladder cancer and abdominal wall-metastasized colon cancer have a poor prognosis despite the advances in surgical techniques, chemotherapy, and radiation therapy. Patients with progressive cancer are waiting for new additional therapies, such as the combination of irradiation and immunotherapy. We have already reported on the success of combined treatments including AFTV for advanced breast cancer with bone metastasis [1]. Therefore, this is the second report of a long-lasting complete response of stage IV cancer after combined treatments including AFTV from our institution.

Gall bladder cancer is an uncommon disease in most parts of the world, despite being the most common and aggressive malignancy of the biliary tree [4]. The management of advanced gall bladder cancer remains a challenge for tumors invading the serosa and/or adjacent organs (T3) and those that invade the main portal vein or hepatic artery, or two or more extrahepatic organs/structures (T4). The benefits of surgical treatment remain unclear compared with potential survival owing to the high rate of surgical invasion; therefore, the therapy should be carefully selected considering the issues with surgery. Surgical resection is only recommended when a curative R0 resection is possible [5]. Given the poor prognosis of gall bladder cancer patients with T ≥ 2 and/or node-positive disease, adjuvant therapy is recommended. For optimal adjuvant therapy, 6 months of gemcitabine- or fluoropyrimidine-based chemotherapy with or without fluorouracil-based chemoradiation can be considered [5]. Most large studies of gall bladder cancer have demonstrated only a 2.7–15% overall 5-year survival rate in the USA and in European countries [6–8]. In a Japanese study on 4774 patients, the 5-year survival rates for stage III, IVA, and IVB cancers were 29, 12.4, and 2.5, respectively [9]. The reason for this reported difference may be the more extensive resection routinely performed by Japanese surgeons, although patient selection bias, differences in pathologic staging, and other variables might have caused this difference in the survival rate [10]. In the context of the case of stage IV gall bladder cancer, it is difficult to mention the real cause of improved survival. However, case 1 was diagnosed with multiple liver metastases and lymph node metastasis, and we should consider that undetectable latent cancer cells spread widely around the primary tumor. Thus, the initial R0 surgery may not have completely removed the entire tumor burden. Therefore, it is highly probable that the combination of AFTV and chemotherapy has been suppressing any recurrence, as confirmed on CT, for more than 5 years.

The case fatality rates for stage I, II, III, and IV colorectal cancers have been reported to be approximately 6, 11, 30, and 79%, respectively [11]. In particular, the 5-year survival rate of synchronous peritoneal carcinomatosis (PC) from colorectal cancer (8.1%) was worse than that of metachronous PC from colorectal cancer (25.4%) [12]. The presence of PC from colorectal cancer is frequently diagnosed during surgery, and 91% of cases of PC from colorectal cancer are not preoperatively detected [13]. The operating surgeon has to select the appropriate treatment strategy, which can include systemic chemotherapy, complete cytoreductive surgery, intraperitoneal chemotherapy, and systemic chemotherapy; temporary surgery, such as a stoma; systemic chemotherapy alone or best supportive care; etc. [14]. In case 2, adjuvant chemotherapy was stopped because of cerebral infarction. The combination of irradiation and immunotherapy with AFTV, as a new additional therapy, did not cause any grade 2–4 adverse effects and ensured long-lasting complete remission. The effect of AFTV could be further explored in other similar cases. In addition, hyperthermic intraperitoneal chemotherapy (HIPEC) surgery is another option for cases in which first-line chemotherapy fails to downstage the cancer [15].

It has been reported that X-ray irradiation upregulates glioma cell immunogenicity [16], and this phenomenon suggests that the combination of irradiation and immunotherapy may be a good therapeutic candidate against malignant cells [17–19]. Moreover, it has been shown that this combination treatment can enhance antitumor effects [20–22]. Active immunotherapy can induce tumor-specific cytotoxic T lymphocytes (CTLs) and achieve a long-term antitumor immune response, and the autologous fixed tumor tissue is expected to provide many tumor antigens that may be recognized by a patient’s immune system resulting in a specific antitumor response [23, 24]. Thus, the treatment course of the present patients with the additional immunotherapy (AFTV) is considered to be well justified.

As delayed-type hypersensitivity (DTH) testing is commonly used to measure specific antitumor cellular immune reactivity, it can be used to evaluate the antitumor cellular immune status immediately before and then after AFTV treatment. The reactivity was found to be positive 2 weeks after the last AFTV injection in both of the present cases. Therefore, the cellular immune response against the carcinoma was induced by the AFTV treatment; however, the effect was weak and slow, and we assume that radiation and chemotherapy contributed to the eradication of the carcinoma with metastasis [1].

Generally, the prognoses of stage IV gall bladder cancer and abdominal wall-metastasized colon cancer are very poor, despite the conventional treatments. At present, the quality of life of our patients is very good, without any recurrence. Moreover, the adverse effects of AFTV were less than grade 2 according to the NCI Common Terminology Criteria for Adverse Events (CTCAE) [25]. Therefore, combination therapy including AFTV should be considered for cases of advanced cancer, although larger-scale randomized and pivotal clinical trials are necessary to confirm the efficacy of AFTV.

In summary, we report the success of combination therapy including AFTV in cases of stage IV heavily metastasized gall bladder cancer and abdominal wall-metastasized colon cancer. Both patients experienced long-lasting, complete remission. Therefore, combination therapy including AFTV should be considered in patients with advanced cancer of the digestive organs.

## Abbreviations

AFTV: Autologous formalin-fixed tumor vaccine
CT: Computed tomography
CTLs: Cytotoxic T lymphocytes
DTH: Delayed-type hypersensitivity
HIPEC: Hyperthermic intraperitoneal chemotherapy
PC: Peritoneal carcinomatosis
